# Artificial intelligence in cardiovascular imaging: enhancing image analysis and risk stratification

**DOI:** 10.1259/bjro.20220021

**Published:** 2023-05-17

**Authors:** Andrew Lin, Konrad Pieszko, Caroline Park, Katarzyna Ignor, Michelle C Williams, Piotr Slomka, Damini Dey

**Affiliations:** 1 Biomedical Imaging Research Institute, Cedars-Sinai Medical Center, Los Angeles, California, USA; 2 Monash Cardiovascular Research Centre, Victorian Heart Institute, Monash University and MonasHeart, Monash Health, Melbourne, Victoria, Australia; 3 Department of Interventional Cardiology, Collegium Medicum, University of Zielona Góra, Zielona Góra, Poland; 4 Division of Artificial Intelligence, Department of Medicine, Cedars-Sinai Medical Center, Los Angeles, California, USA; 5 British Heart Foundation Centre for Cardiovascular Science, University of Edinburgh, Edinburgh, United Kingdom

## Abstract

In this review, we summarize state-of-the-art artificial intelligence applications for non-invasive cardiovascular imaging modalities including CT, MRI, echocardiography, and nuclear myocardial perfusion imaging.

## Introduction

Artificial intelligence (AI) applications continue to permeate into cardiovascular medicine, with the potential to increase workflow efficiency, improve cost effectiveness, and inform decision-making.^1^ In parallel, non-invasive imaging modalities, which have a key role in the diagnosis and management of cardiovascular disease, have seen rapid technological advancements over the past decade. With respect to nomenclature, AI refers to the use of computer systems to perform tasks that normally require human intelligence. *Machine learning* (ML) is a subdiscipline of AI which enables these computer systems to automatically learn to perform a task and improve from experience through exposure to large amounts of data.^2^ *Deep learning* (DL) is a specific type of ML which utilizes artificial neural networks to generate automated predictions directly from input data; in medical image analysis, the most commonly used DL networks are convolutional neural networks. *Explainable AI* refers to ML techniques wherein the results of the learning can be understood by humans, and aims to increase the transparency of ML models.^2^ In this paper, we summarize recent AI applications for non-invasive cardiovascular imaging modalities including cardiac CT, cardiac magnetic resonance (CMR), echocardiography, and nuclear myocardial perfusion imaging (MPI), and focus on the potential of AI for enhancing image analysis and risk stratification.

## Cardiac CT

### Coronary artery calcium scoring CT

Coronary artery calcium (CAC), a direct marker of coronary atherosclerosis and strong predictor of future cardiovascular events, can be readily assessed using non-contrast cardiac CT.^3^ In clinical practice, CAC scoring is performed by experts who manually identify high-density voxels in the coronary arteries. Rapid, automated, AI-enabled CAC scoring has the potential to reduce the workload of physicians and technicians and improve the clinical workflow. Wolterink et al^4^ used a random forest ML model to automatically delineate between coronary and non-coronary calcifications based on position, shape, size, and intensity features. The resultant ML-enabled CAC score showed strongly correlation with expert manual measurements (*r* = 0.94). Recently, several DL applications for automatic CAC segmentation and scoring have emerged. Zeleznik et al^5^ trained a U-Net-based convolutional neural network to quantify CAC scores in close correlation with the manual reference standard (*r* = 0.92) and at a speed of 1.94 s per scan. Notably, the DL-based CAC score had strong predictive value for future cardiovascular events in 20,084 individuals from distinct asymptomatic and chest pain cohorts, independent of clinical risk factors. Other investigators have demonstrated the feasibility of DL-based CAC scoring across a diverse range of cardiac and chest CT scan protocols, including coronary CT angiography,^6^ positron emission tomography CT attenuation correction scans,^7^ lung cancer screening CT,^5,8^ and radiotherapy planning CT in patients with breast cancer.^9^ This greatly expands the range of real-world CT scans in which the presence and extent of coronary artery disease (CAD) can be rapidly and accurately assessed, informing risk stratification and potential initiation of preventative therapy.

**Figure 1.**
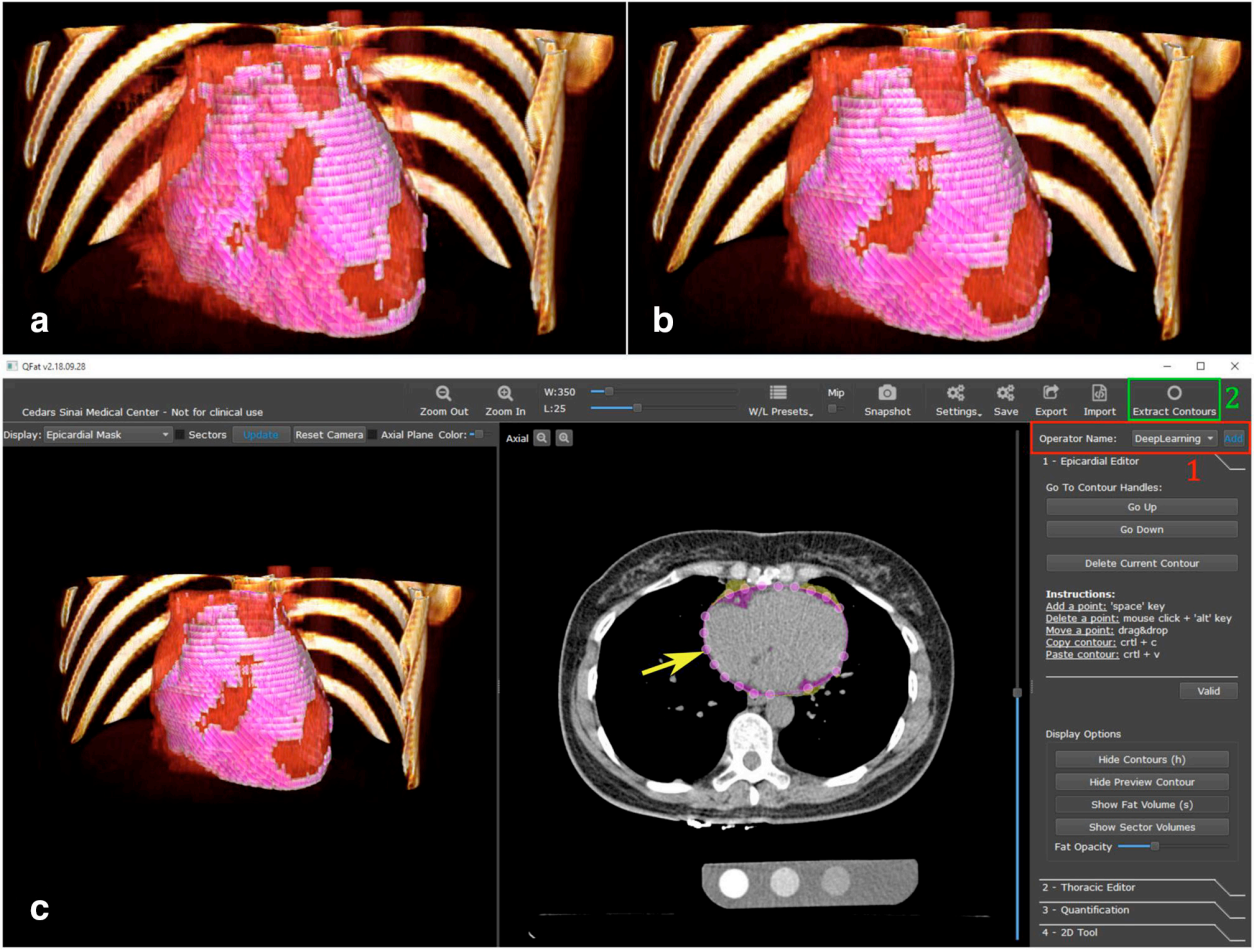
Deep learning-based quantification of epicardial adipose tissue. Three-dimensional rendering of epicardial adipose tissue (pink overlay) derived from coronary artery calcium scoring CT, (**a**) as manually measured by an expert and (**b**) as automatically quantified by a deep learning algorithm embedded in research software (c).

DL applied directly to CAC scoring CT scans allows for automated quantification of additional biomarkers for enhanced outcome prediction. Epicardial adipose tissue, enclosed within the visceral pericardium, is a metabolically active fat depot which has been shown to influence coronary atherosclerotic plaque burden and characteristics.^10^ Commandeur et al^11^ trained a DL algorithm for fully automated EAT quantification, and testing in a large multicenter study and yielded excellent correlation between DL and expert manual measurements (Figure 1).). DL analysis was rapid (6 s per scan) compared to the 15 min taken by experts. In 2068 asymptomatic subjects undergoing CAC scoring CT, DL-enabled epicardial adipose tissue volume and attenuation measurements were predictive of future adverse cardiac events at 14 years, independent of the clinical cardiovascular risk score or CAC score.^12^ This DL algorithm for rapid, high-throughput quantification of epicardial adipose tissue could potentially be implementated into routine reporting of calcium scoring CT, providing additive and complementary information on cardiometabolic risk.

**Figure 2.**
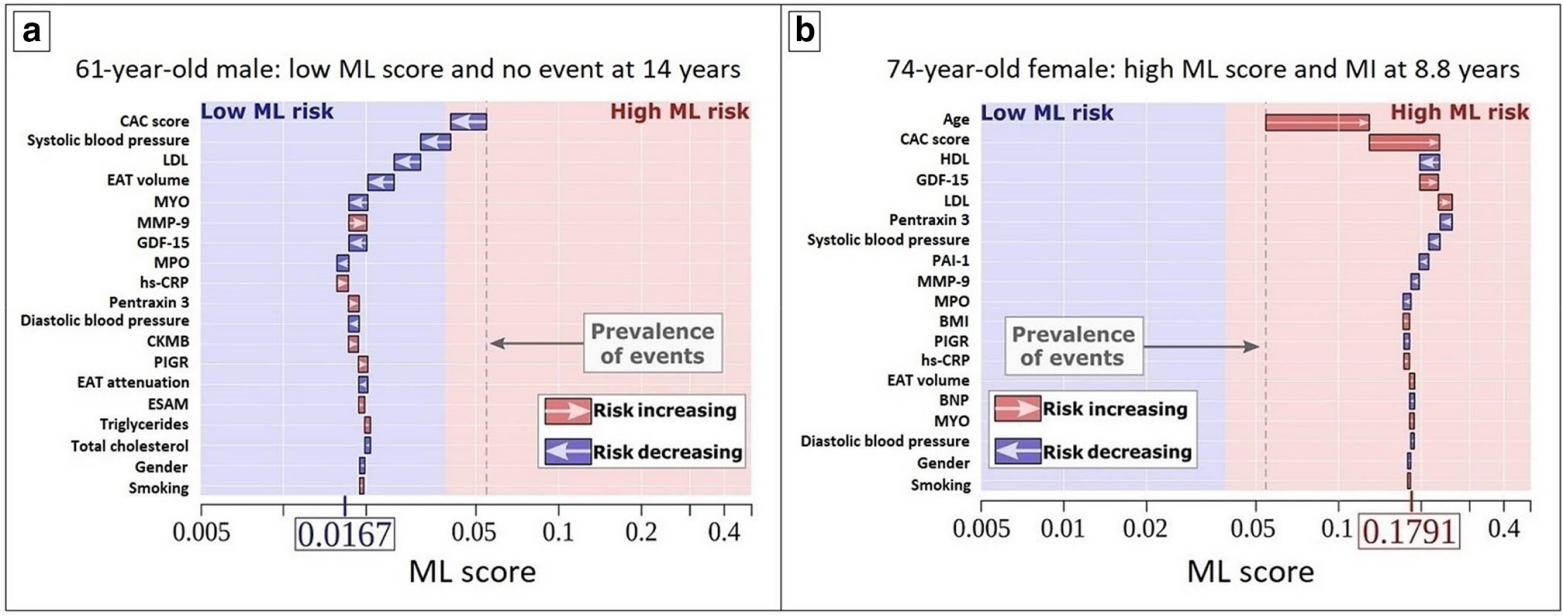
Explainable artificial intelligence through individualized ML risk prediction (**a**) 62-year-old male with no event at 14 years and (**b**) 74-year-old female with an MI at 8.8 years. The X-axis denotes the ML risk score which is the predicted probability of events. The arrows represent the influence of each variable on the overall prediction; blue and red arrows indicate whether the respective parameters decrease (blue) or increase (red) the risk of future events. The combination of all variables’ influence provides the final ML risk score. The subject in (a) has a low ML risk score (0.0167), with an ASCVD risk score of 7.25% and CAC score of 0. The subject in (b) has a high ML risk score (0.1791), with an ASCVD risk score of 30.4% and CAC score of 324. The blue and red background colors indicate low versus high ML risk according to study-specific threshold, and the gray dashed line corresponds to the base risk obtained from the prevalence of events in the population (4.7%). ASCVD, atherosclerotic cardiovascular disease; BNP, brain natriuretic peptide; CAC, coronary artery calcium; CKMB, creatine kinase MB; CRP, C-reactive protein; EAT, epicardial adipose tissue; ESAM, endothelial cell-selective adhesion molecule; GDF-15, growth differentiation factor 15; HDL, high-density lipoprotein; LDL, low-density lipoprotein; MCP-1, monocyte chemoattractant protein 1; ML, machine learning; MMP-9, matrix metalloprotease 9; MPO, myeloperoxidase; PAI-1, plasminogen activator inhibitor 1; PIGR, polymeric immunoglobulin receptor.

Metrics derived from non-contrast CAC scoring CT can also be input into ML models for risk stratification. In a prospective study of 1069 subjects with long-term follow-up, ML integration of clinical risk factors, CT imaging measures (including CAC score and epicardial adipose tissue measurements) and circulating biomarkers (area under the receiver operating characteristic curve [AUC] 0.81) outperformed the CAC score (AUC 0.75) and ASCVD risk score (AUC 0.74) alone (both *p* = 0.02).^13^ Serum biomarkers provided incremental prognostic value beyond clinical data and CT measures in the ML model (net reclassification index 0.53 [95% CI: 0.23–0.81], *p <* 0.0001). A major strength of this ML approach the in-built ability to explicitly describe the contribution of each variable to the final patient-specific prediction and provide explainable AI (Figure 2). In 66,636 asymptomatic individuals from the CAC Consortium, Nakanishi et al^14^ applied a ML classifier integrating the total CAC score with 30 other quantitative CT measures (including aortic valve, mitral valve, and thoracic aortic calcium scores) and 46 clinical features for predicting cardiovascular mortality at 10 years. ML (AUC 0.85) outperformed both the atherosclerotic cardiovascular disease risk score alone (AUC 0.82) and CAC score alone (AUC 0.78; both *p* < 0.001) for outcome prediction.

### Coronary CT angiography

Coronary computed tomography angiography (CCTA) is now a first-line modality for assessing luminal stenosis severity in stable chest pain patients. While this relies on visual estimation by clinicians,^15^ reader experience in a high-volume center is required to grade stenosis with acceptable accuracy.^16^ Automated and objective stenosis evaluation by AI has the potential to reduce interreader variability and interpretative error, and thus several DL-based applications have emerged. Hong and colleagues^17^ employed a M-Net-based convolutional neural network to automatically measure minimal luminal area and percentage diameter stenosis, achieving strong correlation (*r* = 0·984 and 0·957, respectively) with manual expert measurements. Meanwhile, Choi et al^18^ leveraged a series of convolutional neural networks to categorize stenosis severity by CAD-RADS (Coronary Artery Disease–Reporting and Data System) in close agreement with expert consensus (κ = 0.72 per-vessel level and 0·81 per-patient level).

**Figure 3.**
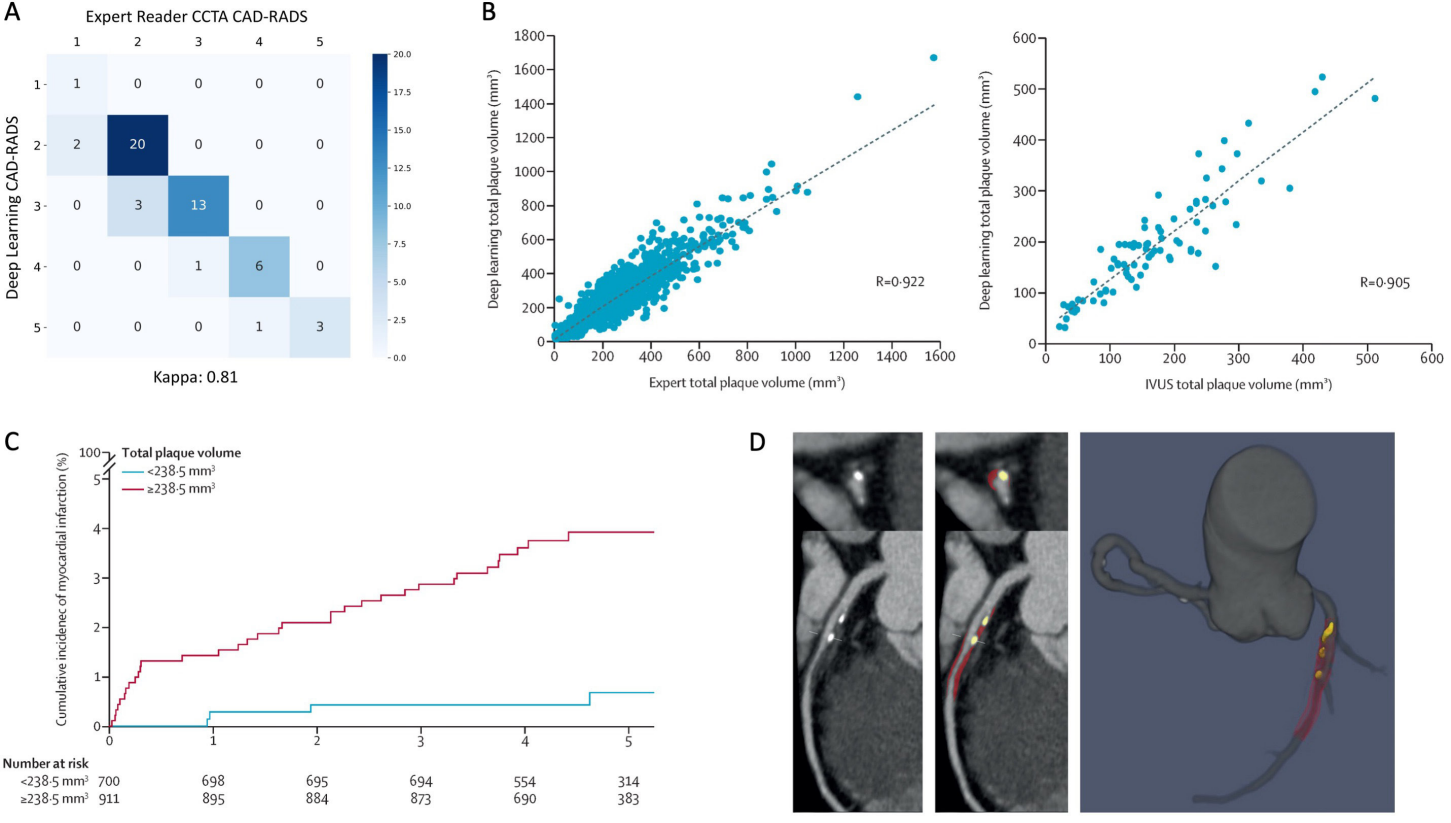
Performance of a DL system for automated measurements and risk prediction on CCTA. (**a**) Confusion matrix of DL *vs* clinician categorization of stenosis severity according to the CAD-RADS. (**b**) Correlation plots of total plaque volume measured by DL *vs* expert readers (1901 lesions) and intravascular ultrasound (IVUS; 84 lesions). (**c**) Kaplan–Meier cumulative incidence curves of myocardial infarction in patients from the SCOT-HEART trial stratified by DL-based total plaque volume ≥238.5 mm^3^, the optimum cut-off determined by receiver operating characteristic curve analysis. (**d**) Case example of DL plaque segmentation in the proximal left anterior descending artery, with curved multiplanar views (left panels) and 3D rendering (right panel) showing calcified plaque (yellow) and non-calcified plaque (red). CAD-RADS, Coronary Artery Disease–Reporting and Data SystemCCTA, coronary computed tomography angiography; DL, deep learning; SCOT-HEART, Scottish Computed Tomography of the HEART).

More recently, a novel DL system (“Hierarchical ConvLSTM”) for automated coronary plaque segmentation was developed and externally validated in an international multicenter study of 1196 patients (6946 lesions).^19^ The DL system demonstrated good agreement with expert readers for quantitative measurements of percent diameter stenosis (intraclass correlation coefficient^20^ 0.88). Further, CAD-RADS categorization of stenosis by DL exhibited close agreement with clinician interpretation of CCTA (κ = 0.81 at the patient level) (Figure 3a) and high diagnostic performance for excluding stenosis ≥70% on invasive coronary angiography (sensitivity 90% and negative-predictive value 94%). The mean DL computation time was 5.7 s per scan *vs* 25.7 min taken by experts. Beyond stenosis assessment, the DL system also computed total plaque volumes for each lesion, yielding excellent agreement with expert reader measurements (ICC 0.96) and intravascular ultrasound (ICC 0.95) (Figure 3b). Importantly, DL-based per-patient total plaque volume was predictive of future myocardial infarction in stable chest pain patients from the prospective SCOT-HEART (Scottish Computed Tomography of the HEART) trial; patients who had total plaque volume ≥238·5 mm^3^ were at greater than five-times risk of myocardial infarction, independent of the presence of DL-based obstructive stenosis and their cardiovascular risk score (Figure 3c). Such a DL system could potentially be implemented in routine CCTA workflow, performing rapid and automated measurements in real-time (Figure 3d) and augmenting clinical CCTA interpretation and risk stratification.

AI techniques have also been applied for the functional assessment of coronary stenosis on CCTA. Non-invasive CCTA-derived fractional flow reserve (CT-FFR) is routinely performed in clinical practice with the aid of DL algorithms which delineate lumen boundaries and thus enable simulation of computational fluid dynamics (CFD).^21^ However, current CFD-based CT-FFR is computationally demanding and requires off-site analysis in core laboratories. To address these limitations, Itu et al^22^ developed a fully automated DL algorithm for the on-site calculation of CT-FFR, which exhibited a sensitivity of 82% and specificity of 85%, referenced by invasive FFR. The DL computation time per-patient was much more rapid than CFD (2.4 *vs* 196.3 s). This DL-based CT-FFR method was then validated in a multicenter study of 351 patients and 525 vessels,^23^ where it improved the diagnostic performance of CCTA and was comparable to conventional CFD-based CT-FFR. This DL application has the potential to facilitate CT-FFR calculation at a standard workstation at the point-of-care.

**Figure 4.**
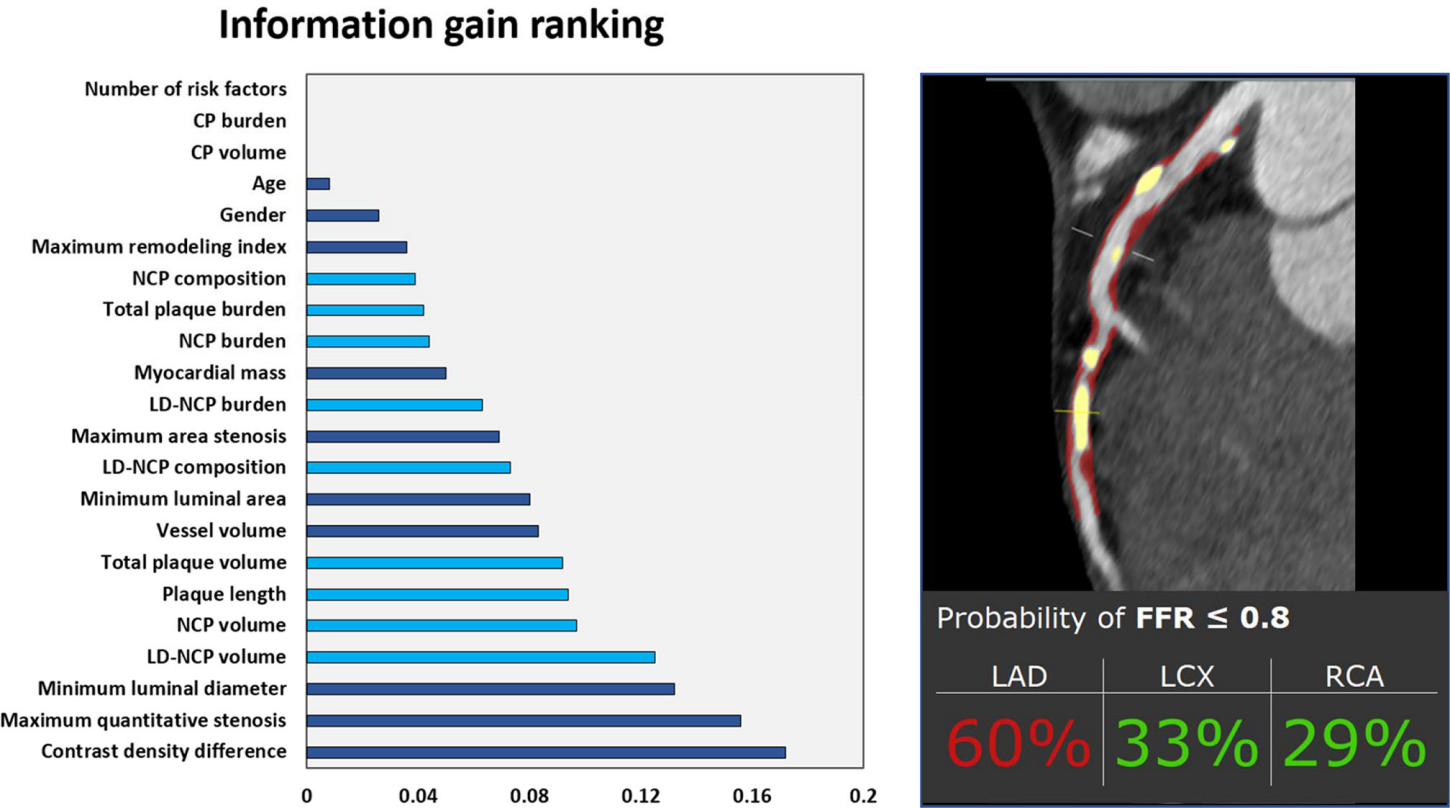
Machine learning prediction of lesion-specific ischemia. Left panel: information gain ranking of variables in a CCTA-based machine learning Ischemia Risk Score to predict lesion-specific ischemia by invasive FFR. Quantitative plaque measures are shown in light blue and other CCTA and clinical variables are shown in dark blue. Right panel: case example of the Ischemia Risk Score applied to a patient, with NCP and CP shown in red and yellow overlay, respectively. Invasive FFR of the LAD was positive (0.73). CCTA, coronary computed tomography angiography; CP, calcified plaque; FFR, fractional flow reserve; LAD, left anterior descending artery; LD-NCP, low-density non-calcified plaque; LCX, left circumflex artery; NCP, non-calcified plaque; RCA, right coronary artery.

Stenosis severity and plaque characteristics derived from CCTA have also been combined using ML models to predict outcomes. Motwani et al^24^ trained a ML classifier integrating 25 clinical variables and 44 visually assessed CCTA parameters of CAD extent, severity, and composition to predict of all-cause mortality at 5 years in 10,030 patients with suspected CAD. The calculated ML score (AUC 0.79) was superior to current CCTA segment scores (AUC 0.64) and the Framingham risk score (AUC 0.61; both *p* < 0.001) . According to feature important, the highest-ranked predictors in the ML model were the number of segments with non-calcified plaque and number of vessels with non-obstructive stenosis. ML models incorporating anatomical plaque measurements can also be used to assess the functional significance of coronary stenosis. In a substudy of the NXT (Analysis of Coronary Blood Flow Using CT Angiography: Next Steps) trial, Dey et al^25^ combined CCTA-derived quantitative plaque parameters using ML to compute a vessel-specific “Ischemia Risk Score”, which was the continuous probability of an abnormal invasive FFR (≤0.80). The resultant ML score had a higher AUC (0.84) than luminal stenosis severity (0.76, *p* = 0.005) or a clinical risk score (AUC 0.63, *p* < 0.001) for predicting an abnormal FFR. The plaque features with greatest contribution to ML preddfiction were non-calcified plaque volume and low-attenuation plaque volume (defined as all plaque-containing voxels <30 Hounsfield units) (Figure 4).

### Heart valves

Cardiac CTA is now integrated into procedural planning for transcatheter valve replacement, enabling 3D native valve modeling of the native valve and device sizing. Grbic et al^18^ used ML algorithms combined with marginal space learning—a 3D learning-based object detection method—to automatically generate individualized models of all four heart valves from 4DCT cine images, trained using expert reader manual annotations as ground truth. For the aortic and mitral valves, differences between ML and expert measurements of valve position and orientation only were slightly above the interreader variability. Meanwhile, Liang and colleagues^17^ showed how a ML algorithm using leaflet meshes could automatically reconstruct patient-specific 3D models of the aortic valve in close agreement with expert manual measurements (landmark-to-mesh distance of 0.69 mm for ML, compared to an interobserver error of 0.65 mm).

Al et al^26^ employed a regression-based ML algorithm to precisely localize eight different aortic valve landmarks at rapid speeds (≤12 ms) on pre-procedural planning CTA for transcatheter aortic valve replacement. In a hybrid imaging approach, Zheng et al^27^ utilized marginal space learning to automatically segment the aorta on rotational C-arm CT images, generating a 3D aorta model which was super-imposed on 2D fluoroscopic images in real-time during live transcatheter aortic valve replacement cases. The resultant patient-specific valve models were then used to assess the biomechanical interaction between the valve bioprosthesis and the surrounding tissue, which has the potential to facilitate device selection improve the likelihood of device success.

### Cardiac MR

**Figure 5.**
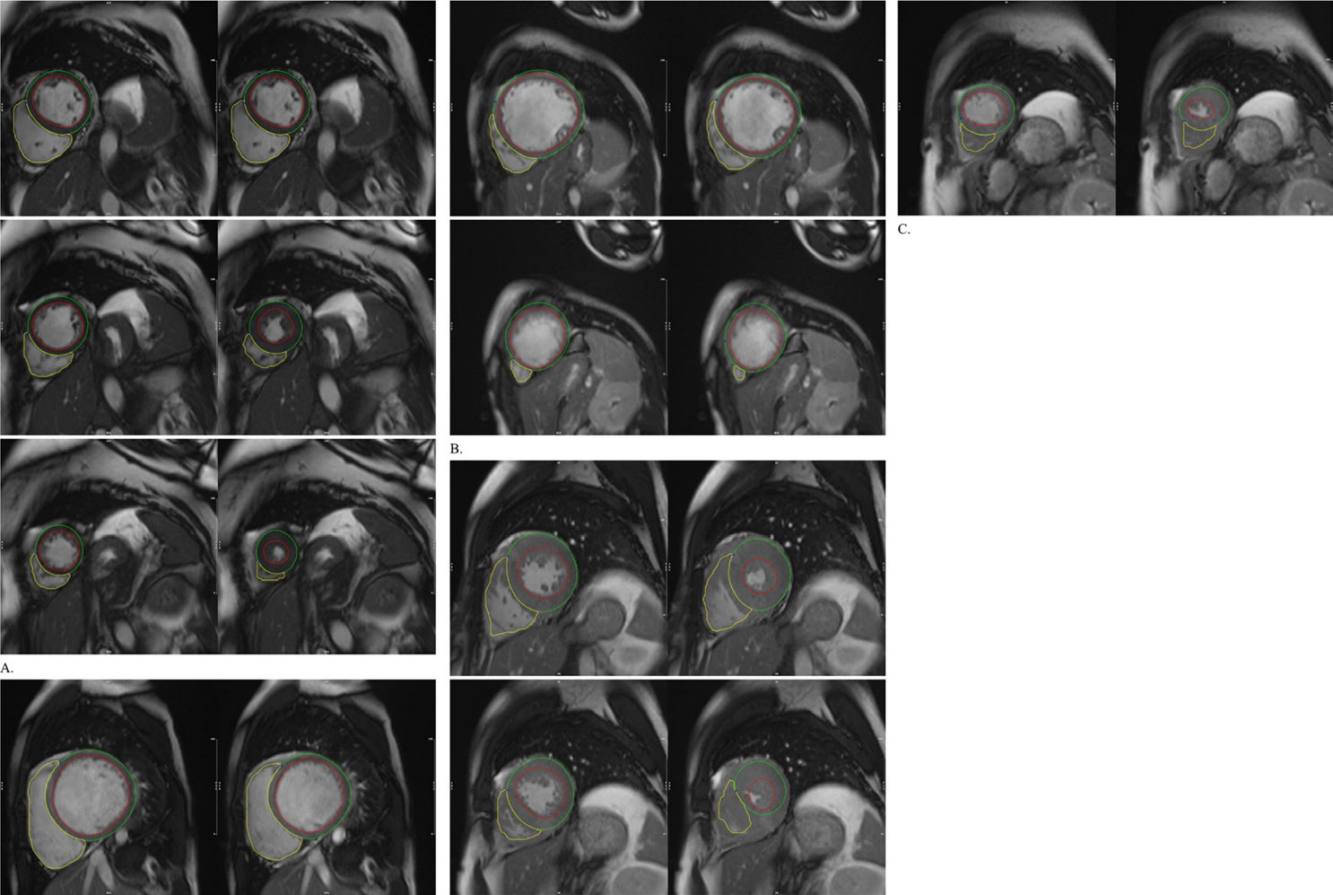
Examples of automated left ventricular segmentation on CMR by deep learning. The basal, mid, and apical sections of the left ventricle are shown in end-diastole (left panels) and end-systole (right panels). The left ventricular endocardial and epicardial contours are denoted by red and green circles, respectively, and the right ventricular endocardial contour is denoted by the yellow circle. (**a**) Normal individuals. (**b**) Patients with dilated cardiomyopathy. (**c**) Patients with hypertrophic cardiomyopathy. Reproduced with permission from Guo et al.^35^ CMR, cardiac MR.

The availability of large public data sets such as the UK Biobank has helped catalyze the proliferation of AI applications in CMR, and there are currently more computational models for image analysis in this domain than in any other cardiac imaging modality. CMR is accepted as the best technique for assessing cardiac structure and global systolic function, through the measurement of absolute blood and myocardial volumes. Image acquisition is standardized and can be delivered in a short time frame, however image analysis is time-consuming and operator-dependent, with inherent intra- and interobserver variability. DL can be used to automate this task with accuracy and precision. In a large data set of 4875 subjects and 93,500 images from the UK Biobank, Bai et al^28^ showed a convolutional neural network to perform comparably to human experts for the segmentation of all four cardiac chambers on cine images, with a Dice coefficient (measuring overlap between DL and manual segmentations) ranging from 0.90 to 0.96. Various DL-enabled fully automated segmentation algorithms have achieved excellent agreement with expert readers in performing measurements of left and right ventricular volumes and ejection fractions.^29–33^ Further, the scan-rescan reproducibility of DL-enabled cardiac structure and function measurements was recently demonstrated by Davies et al,^34^ whose U-Net-based convolutional neural network outperformed three clinicians (scan-rescan coefficient of variation for DL *vs* clinician: left ventricular ejection fraction 4.2 *vs* 6.0%; left ventricular mass 3.6 *vs* 4.8%) in a fraction of the analysis time (20 s *vs* 13 min). Importantly, the performance of DL for left ventricular segmentation can be affected by various disease states, with a recent study demonstrating stronger correlation between DL and manual measurements of ejection fraction in hypertrophic versus dilated cardiomyopathy (*r* = 0.94 *vs* 0.88)^35^ (Figure 5).

AI has also shown promise in CMR tissue characterization. Left ventricular scar volume on late gadolinium enhancement imaging is an important biomarker in risk stratification and procedural planning. DL convolutional neural networks can delineate left ventricular scar from normal myocardium using image-based features, enabling accurate quantification of scar volume in patients with ischemic^36^ and hypertrophic^37^ cardiomyopathies. Fully automated DL-based techniques have also been developed to measure the burden of left atrial scar in patients with atrial fibrillation, potentially helping to inform patient selection for catheter ablation.^38,39^ Native T1 mapping on CMR enables the quantification of diffuse myocardial fibrosis, and a DL algorithm which automatically measures global and regional myocardial T1 values has demonstrated strong agreement with expert manual measurements.^40^

CMR biomarkers can also be incorporated into ML models to enhance risk prediction. Ambale-Venkatesh et al^41^ used a random forest algorithm to show that among 725 clinical and imaging variables, CMR-derived left ventricular structure and function were highly ranked predictors of incident heart failure in 6814 subjects from the MultiEthnic Study of Atherosclerosis. The ML model prediction surpassed established risk scores and had higher prediction accuracy. In survivors of myocardial infarction, Kotu et al^42^ employed a ML model integrating CMR-derived scar characteristics such as size, location, and texture features to predict the occurrence of cardiac arrhythmias. This resultant AUC of 0.92 for ML was comparable to currently used criteria such as left ventricular ejection fraction. Finally, Bello et al^43^ trained a DL classifier using 3D models of right ventricular motion on CMR to accurately predict survival in patients with pulmonary hypertension; outperforming conventional statistical Cox models containing right ventricular volume and strain parameters.

### Echocardiography

With rapid image acquisition, relatively low cost, and lack of ionizing radiation, echocardiography remains the most widely used cardiovascular imaging modality. Large, well-structured and labeled data sets such as the National Echocardiography Database Australia^44^ have been established and are well-primed for AI applications.

A fundamental metric of cardiac function is left ventricular ejection fraction, which determines selection of drug therapy in heart failure,^45^ detects cancer therapy-related cardiac dysfunction,^46^ acts as a gatekeeper for cardiac devices, and provides a surrogate endpoint in drug development and outcome prediction.^47,48^ Current human assessment of ejection fraction requires tracing of left ventricular end systolic and end diastolic volumes which can lead to considerable interobserver variability.^49^ There has been great interest in using DL techniques for echocardiography to determine ejection fraction. View classification, the essential first step in the process, was automated by Madani et al,^50^ who designed a convolutional neural network which could classify 15 standard views on transthoracic echocardiograms with 91.7% accuracy, compared with 70.2–84.0% for board-certified echocardiographers. Similarly, Zhang et al^51^ trained a DL network using 14,035 echocardiograms to identify 23 standard views and flag partially obscured cardiac chambers. Ghorbani et al^52^ developed a DL model for the prediction of left ventricular volumes and ejection fraction from sampled apical-four-chamber view images, achieving R^2^ scores of 0.70, 0.74, and 0.50 for end-diastolic volume, end-diastolic volume, and ejection fraction, respectively, compared to clinician measurements. However, these described DL approaches rely on manually curated still images at systole and diastole instead of using the actual echocardiogram videos. Ouyang et al^53^ proposed a video-based DL algorithm (EchoNet-Dynamic) for beat-to-beat estimations of left ventricular ejection fraction. Trained on 10,030 apical-four-chamber echocardiogram videos, the convolutional network identified each cardiac cycle, generated a clip of 32 frames, and averaged estimates of the ejection fraction for each beat. EchoNet-Dynamic achieved Dice coefficients of 0.93 and 0.90 for end-diastolic and end-systolic volumes, respectively, referenced by human experts. For the prediction of ejection fraction, the algorithm had an R^2^ of 0.81 and mean absolute error of 4.1% compared with expert annotations, and reliably classified heart failure with reduced ejection fraction (AUC of 0.97).

3D echocardiography is now the recommended over 2D echocardiography for cardiac chamber quantification due to its superior accuracy and reproducibility.^54^ However, 3D quantification is time-consuming and requires expertise, limiting its integration into clinical practice. ML techniques can automate this process, providing rapid and reproducible measurements of left ventricular and left atrial volumes which are comparable to CMR reference values^55,56^ and generalizable to external centers^57^ and patient populations.^58^ Further tuning of these ML algorithms has enabled the calculation of LV mass^59^ and right ventricular volume and ejection fraction.^60^ Using AI-based software to analyze 3D echocardiography may improve workflow efficiency as more institutions adopt this technology.

Echocardiography also enables quantification of cardiac structure and identification of morphological abnormalities in different disease states. Left ventricular wall thickness measurement, a marker of the degree of left ventricular hypertrophy, is subject to substantial intra- and interobserver variability.^61^ Moreover, the etiology of increased left ventricular wall thickness is often difficult to determine on routine echocardiographic images by human observation. To address these limitations, Duffy et al^62^ trained a DL algorithm to measure left ventricular wall thickness on echocardiogram videos with high accuracy when tested in an external data set (mean absolute error 1.7 mm and 1.8 mm for the intraventricular septum and posterior wall, respectively, compared with expert measurements). This DL algorithm also accurately classified cardiac amyloidosis (AUC 0.79) and hypertrophic cardiomyopathy (AUC 0.89) separately from other causes of left ventricular hypertrophy.

With respect to cardiovascular risk stratification, Narula et al^63^ trained a ML model integrating 20 different echocardiographic variables acquired at end systole to outperform conventional diastolic and strain parameters in differentiating physiological from pathological patterns of left ventricular hypertrophy. Lancaster et al^64^ employed ML hierarchical clustering of diastolic echocardiographic parameters for the classification of diastolic dysfunction, with ML clusters providing improved prediction of event-free survival over guideline-based classification.^65^ Recently, Kobayashi et al^66^ used K-means clustering of clinical, biochemical, and echocardiographic parameters to identify three distinct phenotypic groups (“mostly normal”, “diastolic changes”, and “diastolic changes with structural remodeling”), each with different long-term risk of heart failure hospitalization or cardiac death. These studies demonstrate the potential of such ML techniques for grouping directly observed echocardiographic parameters and uncovering patient phenotypes with a similar behavior (a latent, or hidden, state) for predicting future adverse events. Such algorithms could help idenfity patients with high-risk phenotypes who may benefit from more careful follow-up or targeted heart failure therapies.

### Nuclear myocardial perfusion imaging

The domain of nuclear cardiology has seen rapid advancements in AI applications in recent years. Single-photon emission computed tomography (SPECT) MPI is widely used for patients with suspected CAD. Total perfusion deficit (TPD) on stress MPI is a standardized quantitative measurement of the extent and severity of myocardial ischemia which has similar accuracy to clinical visual interpretation for detecting obstructive stenosis.^67^ Arsanjani et al^68^ used a ML classifier to combine MPI parameters (including stress TPD and stress-induced ECG changes) with clinical variables for the diagnosis of ≥70 stenosis on invasive coronary angiography. The resultant ML model (AUC 0.94) outperformed stress TPD alone (AUC 0.89) and clinician interpretation (AUC 0.85; both *p* < 0.0001). MPI data have also been incorporated into ML models for outcome prediction. In an analysis of REFINE-SPECT (Registry of Fast Myocardial Perfusion Imaging with NExt generation SPECT), a collaborative international multicenter registry of over 20,000 patients undergoing stress MPI, Hu et al^69^ used ML to combine quantitative measures of regional perfusion deficits with stress test and clinical variables to predict early coronary revascularization. They showed ML prediction to outperform both clinician interpretation and stress TPD assessment. Similarly, Betancur et al^70^ employed a ML classifier for predicting 3 year cardiac event risk in 2619 patients undergoing stress MPI. The ML model integrating imaging, stress test, and clinical parameters was superior to clinician interpretation, TPD assessment, or ML with only imaging variables (AUC 0.81 *vs* 0.73 *vs* 0.71 *vs* 0.65; *p* < 0.01 for all) for predicting events. Moreover, the comprehensive ML model provided a 26% risk reclassification for events compared to clinician interpretation. These studies highlight the role of ML in selecting and ranking the most important predictor variables, allowing clinicians and researchers to interpret the relative influence of each variable on individual patient risk.

**Figure 6.**
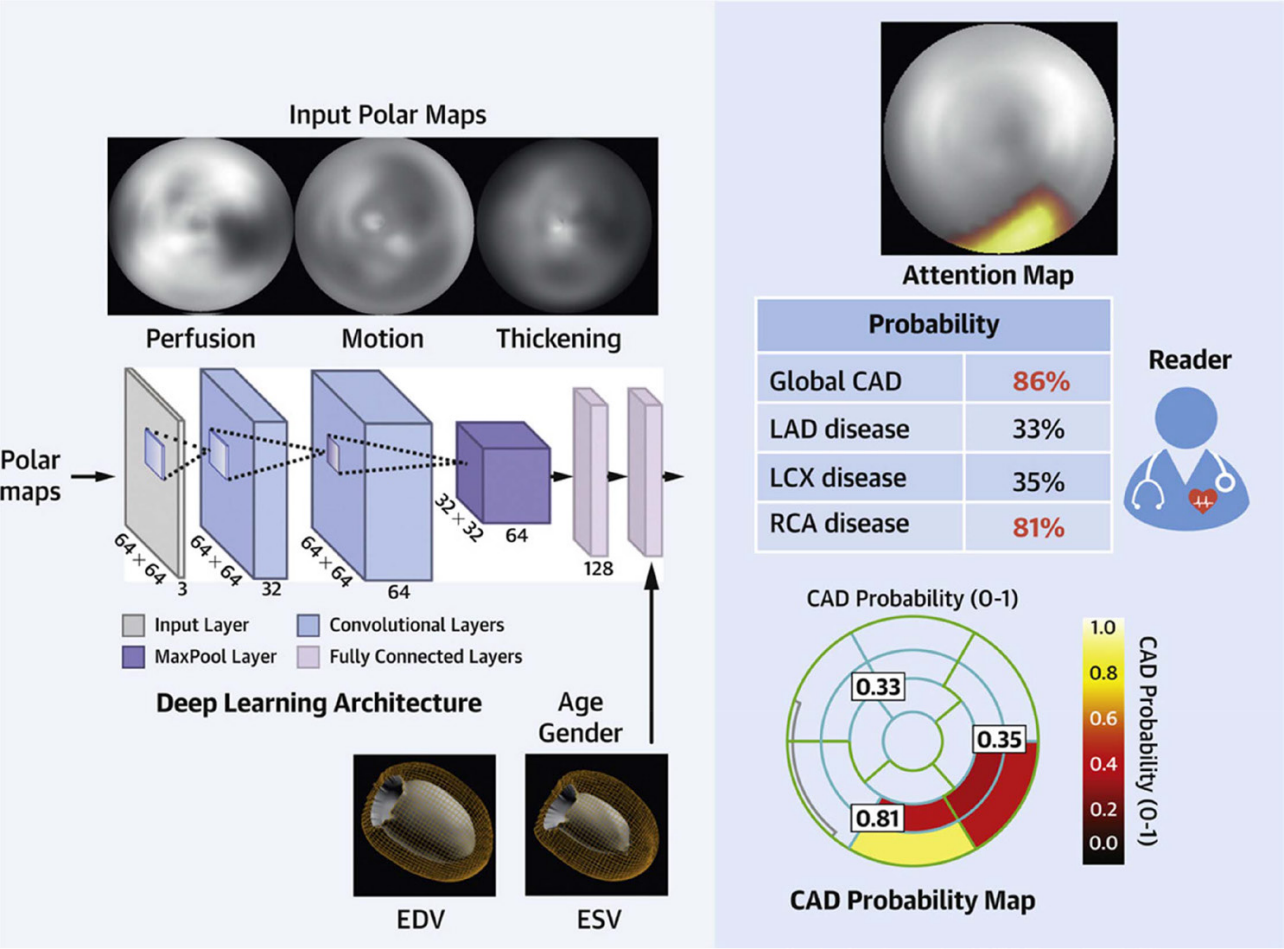
Clinical implementation of explainable AI for detecting obstructive CAD on SPECT MPI. Left panel: architecture of the DL model, which calculated the probability of obstructive CAD using stress myocardial perfusion, wall motion, and wall thickening maps, as well as left ventricular volumes, age, and sex. Right panel: attention maps highlighting myocardial regions (coronary artery territories) contributing to the DL prediction were generated to the explain the findings to clinicians. AI, artificial intelligence; CAD, coronary artery disease; DL, deep learning; EDV, end-diastolic volume; ESV, end-systolic volume; LAD, left anterior descending artery; LCX, left circumflex artery; RCA, right coronary artery; SPECT MPI, single-photon emission computed tomography myocardial perfusion imaging.

DL algorithms can perform direct, automated analysis of MPI images for diagnosis and prognostication of outcome in CAD. In 1638 patients from REFINE SPECT, Betancur et al^71^ trained a convolutional neural network using standard polar map images to predict obstructive coronary stenosis. The DL prediction exhibited a higher AUC than TPD assessment (0.80 *vs* 0.78, *p* < 0.01) and was computed at a speed of <0.5 s per scan. In a pragmatic study, Otaki et al^72^ implemented an explainable AI model for the detection of obstructive CAD on SPECT MPI into routine clinical reporting software. The DL algorithm automatically calculated the probability of obstructive CAD using stress myocardial perfusion, wall motion, and wall thickening maps, as well as left ventricular volumes, age, and sex (Figure 6). For clinical interpretability, attention maps were generated to highlight myocardial regions (coronary artery territories) contributing to the DL prediction. When tested on 3578 patients with suspected CAD undergoing SPECT MPI at nine centers, the DL algorithm exhibited a higher AUC (0.83) than stress TPD (0.78) or clinician interpretation (0.71; both *p* < 0.0001). DL results were generated in real-time (<12 s on a standard workstation) and the attention maps facilitated the acceptance of AI results by clinicians. Subsequently, Singh et al^73^ applied this DL algorithm to polar maps of 20,401 patients from the comprehensive REFINE SPECT clinical-imaging database for the direct prediction of future adverse cardiac events at 4.4 years. Here, DL achieved an AUC of 0.75 compared with 0.70 (*p* < 0.0001) for stress total perfusion deficit, and patients in the highest quartile of DL-predicted risk were at 10-times greater hazard of a cardiac event compared with patients in the lowest quartile of DL-predicted risk (9.7% *vs*  0.9%).

## Conclusion

Non-invasive cardiovascular imaging contains a wealth of high-dimensional data and is well-suited to AI applications. ML and DL algorithms are increasingly being employed for tasks such as image segmentation, automated measurements, diagnosis, and outcome prediction. Ongoing rapid advancements in computing hardware and software will facilitate the future implementation of these algorithms into clinical workflow. The ultimate goal would be for AI algorithms to be embedded into routine cardiac image analysis and reporting software, automatically performing measurements and calculating risk scores in real-time to function as clinical decision support tools for physicians. Explainable AI will enable clinicians and researchers to interpret ML models and interact with the outputs for an individual patient prediction.
